# Changes in Spine Alignment and Postural Balance After Breast Cancer Surgery: A Rehabilitative Point of View

**DOI:** 10.1089/biores.2018.0045

**Published:** 2019-07-30

**Authors:** Massimiliano Mangone, Andrea Bernetti, Francesco Agostini, Marco Paoloni, Francesco A. De Cicco, Serena V. Capobianco, Arianna V. Bai, Adriana Bonifacino, Valter Santilli, Teresa Paolucci

**Affiliations:** ^1^ Department of Anatomical and Histological Sciences, Legal Medicine and Orthopedics, Sapienza University of Rome, Rome, Italy.; ^2^ Breast Diagnosis and Treatment Unit, Sapienza University of Rome, Sant'Andrea Hospital, Rome, Italy.; ^3^ Umberto I University Hospital, Rome, Italy.

**Keywords:** balance, breast cancer, dysfunction, posture, rasterstereography, stabilometry

## Abstract

Breast cancer is the most common malignant tumor in female patients in developed countries. Recent articles indicate that one-sided mastectomy or minor breast surgery to treat breast cancer can have deleterious effects on posture and the musculoskeletal system. The purpose of this study was to investigate the alterations post-breast cancer surgery of the spine alignment associated to the balance not reported by the noninvasive instrumentation. We enrolled 30 women who had undergone treatment for breast cancer (BG) and were on a waiting-list for rehabilitation treatment and a control group of 30 healthy volunteer women (CG), matched by age and body mass index. The stabilometry was performed using a force platform (Kistler Instruments, Winterthur, Switzerland) test during quiet standing with closed-eyes (EC) and open-eyes (EO), recording the position of the center of pressure (CoP) for 51.2 sec. The stabilogram or the time plot of the two coordinates, X and Y, of the CoP was obtained, which represent anteroposterior and midlateral balance. Spinal posture was measured using the Formetric-4D rasterstereographic system (DIERS, International GmbH, Schlangenbad, Germany), and thoracic kyphotic angle, lumbar lordotic angle, and surface trunk rotation were evaluated. Sixty participants were analyzed (CG:30; BG:30). For the spine rasterstereography a statistically significant difference was shown with regard to anterior–posterior flexion of the trunk major in BG; pelvic inclination and twist of half-pelvis decreased in BG; normalized lumbosacral inversion point decreased in BG; surface rotation major in BG; and lateral deviation major in BG. Compared with the values for the stabilometry test with EO and EC, a statistically significant difference was observed, respectively, for ellipse length (mm; *p* = 0.04) and ellipse area (mm^2^; *p* = 0.04) with EO and in ellipse area (mm^2^) with EC (*p* = 0.05), increased in BG for both conditions. No difference was shown for CoP velocity and oscillations between the groups. Breast cancer survivors after prostheses or tissue expanders for mastectomy showed a spine's misalignment present both on the sagittal plane, both on the coronal and frontal plane, increased in BG regard to anterior–posterior flexion of the trunk, surface rotation, and lateral deviation. It is associated with greater energy expenditure for the postural balance control increased in BG with a major ellipse area in EO and EC conditions and major ellipse length in EC condition.

## Introduction

Breast cancer is the most common malignant tumor in female patients in developed countries.^1^ Although treatment depends on the clinician indications,^2,3^ the primary therapeutic methodology consists of surgery mastectomy or breast-conserving therapy. Recent articles indicate that one-sided mastectomy or minor breast surgery to treat breast cancer can have deleterious effects on posture and the musculoskeletal system such as alterations in spine alignment, an increased thoracic kyphosis and upper limb dysfunctions, and decrease of shoulder joint angles at the operated side,^4^ but other studies appear to contradict these statements.^1,5^ After breast cancer surgery, patients have reduced physical participation in valuable activities contributed, more specifically, to explaining variability in depression.^6^ The patient tends to assume an attitude of closure, especially versus the presurgery “protective posture.” In the early stages after surgery, this attitude is also due to modesty and shyness.

Women who undergo mastectomy alone, compared with women who undergo immediate breast reconstruction with abdominal flaps, for example, show differences in the vertical alignment of the trunk, with greater asymmetry between the acromion and greater trochanter, which can cause trunk rotation.^7–11^ The surgery technique seems to make a difference after breast cancer surgery. Also, lymphedema appears to worsen asymmetries and modifications in posture after mastectomy.^12^ Moreover, the literature is lacking in studies on posture balance after breast cancer surgery by stabilometry, instead a deterioration in bone strength and balance performance after breast cancer treatment can result in injurious falls: Fong et al. showed that Qigong may be a suitable exercise for improving the balance performance and balance self-efficacy.^13^ Postural stabilometry studies that have been performed after reductive or additive mammoplasty indicate. Mazzocchi et al.^14,15^ demonstrated that the head center of mass showed a significant variation on mediolateral direction, which indicated retro-positioning of the head. Some researchers believe that the increased weight of the breasts causes several spinal postural alterations, such as dorsal kyphosis and anterior shoulder dislodgement.^16^

Therefore, from these premises, it is possible to hypothesize that after mastectomy for breast cancer, postural spine alteration may occur especially on the sagittal plane together with a postural imbalance.

Then, the purpose of this study was to investigate whether breast cancer surgery results in spine postural alterations and postural imbalance to compare breast cancer patients with a population of healthy women. The primary outcome was the sagittal spine alignment regarding lumbar lordotic angle (LLA).

## Materials and Methods

### Study design and population

We conducted a case–control observational study consisting of a biomechanical evaluation of postural changes, as assessed within the sagittal and frontal planes, in female patients after surgical treatment for breast cancer and compared the postural balance control in the quiet stance. We enrolled a group of 30 women who had undergone treatment for breast cancer (BG) and were on a waiting list for rehabilitation treatment and a control group of 30 healthy volunteer women (CG), matched by age and body mass index (BMI). Participants were enrolled from January 1, 2018 to June 30, 2018 at the rehabilitation outpatient clinic of Policlinico Umberto I, Sapienza University of Rome (Italy).

All participants (patients and healthy controls) signed an informed consent form, after receiving detailed information about the study aims and procedures as per the Declaration of Helsinki. The protocol study was approved by the Ethics Committee of Sapienza University of Rome and was developed in accordance with the STROBE guidelines.^17^

The inclusion criteria for BG were as follows: total mastectomy performed within 12 months before recruitment (chronic phase) in waiting list for rehabilitative treatment, age from 18 to 60 years, BMI <30, no cognitive dysfunction,^18^ and use of breast prostheses or tissue expanders after mastectomy. The patients started the rehabilitative treatment within 1 month from the physiatric and postural test evaluations.

The exclusion criteria were as follows: conservative surgery, presence of lymphangitis or mastitis, surgical complications after the intervention, neurological deficits and complications, important shoulder joint problems before the intervention for breast cancer, previously diagnosed postural problems (scoliosis >10° Cobb angle), severe lymphedema and web axillary syndrome,^11^ visual problems that were not corrected by lenses, reconstruction with abdominal flaps or latissimus dorsi flaps,^9–11,19^ diabetes, hypertension that was not controlled by drugs, and antidepressant drug use.

The healthy group consisted of volunteer women who were in contact with our rehabilitation center. Their inclusion criteria were age from 18 to 60 years, BMI <30, and no cognitive dysfunction. The exclusion criteria were postural problems, shoulder joint dysfunction, neurological or cognitive impairments, visual problems that were not corrected by lenses, oncological disease, rheumatological disorders, and pregnancy.

## Measurements

All patients and healthy volunteers took part in a physiatrist visit to collect clinical data and measure the main postural parameters, to exclude scoliosis and other postural disorders. If necessary, an X-ray of the spine was obtained.

On the operated side, the physiatrist performed a clinical evaluation of the shoulder joint range of motion (ROM; 1. flexion, 2. extension, 3. adduction, 4. abduction, 5. internal rotation, and 6. external rotation)^20^ according to the scale of the Medical Research Council Manual Muscle Testing (MRC).^21^ A grade of 5/5 on the MRC scale indicates that movement is possible against maximum resistance; 4/5 indicates movement that is possible only against minimum resistance; 3/5 indicates movement that is possible only against gravity; 2/5 indicates movement that is possible only in the absence of gravity; 1/5 indicates evidence of movement; and 0/5 indicates no movement.

### Biomechanical evaluation

#### Stabilometry assessment

Data were collected on a stabilometric platform (software Sway) to measure oscillations, sway area, length, and velocities of center of pressure with closed-eyes (EC) and open-eyes (EO). The stabilometry test was performed during quiet standing in both conditions (EC and EO) for 51.2 sec. After receiving information about the test procedure, the patients and healthy controls were instructed to stand erect, but not at attention, with their arms along the trunk, their feet at an angle of ∼30° open toward the front, and their heels aligned along the mediolateral direction. All tests were performed by the same examiner; thus, the participants were supplied with the same instructions before each test. Three tests were conducted for each trial condition (EO and EC), and we have reported the average scores of the tests. In the EO condition, subjects fixated on a mark on a wall 1.5 m away at eye level. The test order, EO-EC or EC-EO, was randomized. To minimize external disturbances and cues for the test subjects, the environment was brightly lit naturally and quiet.^22^

#### Spine rasterstereography (Formetric)

Spinal posture was measured using the Formetric 4D rasterstereographic system (DIERS, International GmbH, Schlangenbad, Germany). This device projects on the patient's back a series of parallel light stripes that are emitted by a slide projector. A three-dimensional reconstruction of the back surface is generated using triangulation equations, by transforming the stripes and their corresponding curvature into a scatter plot. The back surface curvature was evaluated, and concavity (right lumbar dimple or DR and left lumbar dimple or DL) and convexity (Vertebra prominens or VP) area were detected without reflective markers.^23^ Vertebral rotation in adolescent idiopathic scoliosis was calculated by radiograph and back surface analysis-based methods: correlation between the Raimondi method and rasterstereography. VP and DR and DL are specific back surface landmarks that are recognized automatically with a standard deviation (SD) of ±1 mm for creating a Cartesian coordinate system. For Guidetti et al.,^24^ no reflective markers were positioned on the patients.

**FIG. 1.**
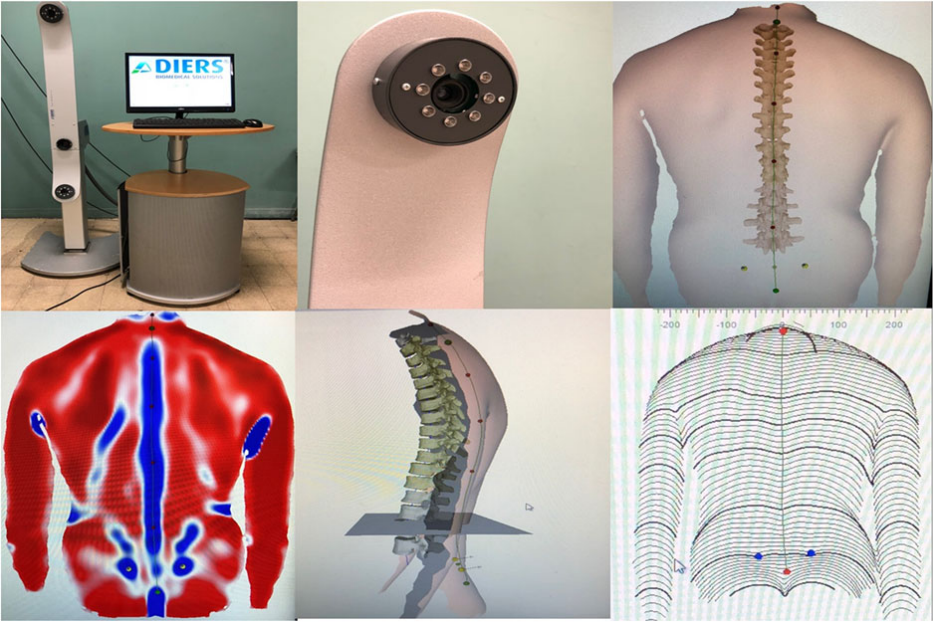
Formetric.

Subjects were placed in a standing position at distance of 2 m from the system, barefoot in comfortable position, with their knees extended and their arms resting naturally alongside their hips. To standardize the subjects' positioning, a horizontal line was drawn on the floor to provide a reference for their heels (Fig. 1).

The following postural parameters were measured: anterior–posterior trunk flexion (mm), lateral trunk flexion (mm), pelvic inclination (mm), twist of half-pelvis (degree), pelvis rotation (degree), apex of dorsal kyphosis (mm), thoracolumbar inversion point (mm), apex of lumbar lordosis (mm), lumbosacral inversion point (mm) normalized, cervical arrow (Stagnara; mm), lumbar arrow (Stagnara; mm), thoracic kyphotic angle (max; degree), lumbar lordotic angle (max; degree), positive surface trunk rotation (+max = right; degree), negative surface trunk rotation (−max = left; degree), and the surface total trunk rotation at the end (amplitude = total; degree).

The trunk inclination (mm) is specified as the plumb line deviation from the VP to the midpoint between dimples (DM) along the sagittal plane; the kyphosis angle (degree) is measured as the angle between tangents of the spine curve, calculated at the points of cervicothoracic and thoracolumbar (ITL) inflexions; the lordotic angle (degree) is measured as the angle between tangents of the spine curve, calculated at the points of the ITL and lumbosacral junction inflexion; the Flèche cervicale and lombaire (mm), or cervical arrow and lumbar arrow, are measured as the distances of the apex of the cervical and lumbar lordosis, respectively, from a virtual vertical plumb line; the pelvic tilt (degree) is calculated as the arithmetic mean between the two angles that are formed by the perpendicular to the surface in the DR and DL to the vertical axis (pelvic torsion average).

### Scales: Functional assessment

The Disabilities of Arm, Shoulder, and Hand Questionnaire^25^ is a 30-item, self-reported questionnaire that is designed to measure physical function and symptoms for disorders of the upper limb to quantify general disabilities that are related to the arm. The items are associated with the degree of difficulty in performing various functional activities due to arm, shoulder, or hand impairments (21 items); the severity of pain, activity-related pain, tingling, weakness, and stiffness (5 items); and the effect on social activities, work, and sleep and its psychological impact (4 items). The total score is converted to a scale from 0 to 100 (100 = greatest disability, 0 = no disability).^26^

The Constant–Murley score is one of the most widely used, valid, and reliable outcome measures for the assessment of the shoulder.^27^ This scoring system consists of subjective variables, such as pain (15 points), activities in daily living (10 points), and arm positioning (10 points), as well as objective variables, such as range of motion (40 points) and strength (25 points). The total score ranges from 0 to 100, with a score of 100 indicating no limits.

### Pain

The visual analog scale is a simple, robust, sensitive, and reproducible instrument that enables patients to express their pain intensity as a numerical value from 0 to 10. Patients are asked to associate the severity of their upper limb pain on the side of surgery with a position on a 10-cm continuous line, marked “no pain” on one end and “worst pain” on the other.^28,29^

### Data analysis

For the sample size calculation, the G * Power Version 3.1.9.2 program was used. The difference between the two groups with respect to the LLA for spine rasterstereography was considered a primary parameter for postural outcome.^30^

The following values were considered for the lumbar lordotic angle with respect to the two groups: mean (BG) = 151.38 (±5.97), mean (CG) = 155.70 (±5.42); for a type 1 error (α) of 5%, a type 2 error (β) of 10%, and a power level of 0.90 (using the G * Power Version 3.1.9.2), the required sample size was 30 participants per group. To allow for possible dropouts from the BG, we enrolled a total of 60 participants (30 patients in BG and 30 in CG).

The descriptive data were presented as means and SDs for all continuous variables. Variables were tested for normality using Shapiro–Wilk test; all the outcome measures were not normally distributed and so Mann–Whitney *U*-test was used to detect difference between groups. The significance level was set at *p* < 0.05. Data were analyzed using MedCalc 12.2.1.0 (MedCalc Software).

## Results

Data from 60 participants were analyzed: 30 participants in the CG (mean age 48 ± 4.80 years with a BMI of 24 ± 1.20) and 30 patients in the BG (mean age 50 ± 5.94 years with a BMI of 24 ± 0.70). The descriptive data of the sample, perfectly matched for age and BMI, are shown in Table 1.

**Table 1.** Clinical Parameters

Clinical parameters	Control group (*n* = 30)	Breast cancer group (*n* = 30)
Age (mean ± SD)	48 ± 4.80	50 ± 5.94
BMI (mean ± SD)	24 ± 1.20	24 ± 0.7
Married/common-law wife	80%	73%
Working	Working 83%	Working 68%
Not employed	Not employed 11%	Not employed 20%
Or retired from work	Retired 6%	Retired 12%
At least a high school education	30%	38%
Clinical characteristics		
Chemotherapy	—	34.7%
Radiotherapy	—	55.4%
Mild Lymphedema	—	10%
Time from surgery (months)	—	4.65 ± 3.30
Scale scores for shoulder and upper limb disability		
DASH scale	—	58 ± 14.4
CMS	—	61 ± 8.62
VAS	—	2.54 ± 2.46

Descriptive data (mean and SD) of the sample, matched for age and BMI. Control group (healthy) and breast cancer group (BG).

BMI, body mass index; CMS, Constant–Murley score; DASH, Disability of the Arm, Shoulder, and Hand; SD, standard deviation; VAS, visual analog scale.

For the spine rasterstereography, a statistically significant difference was shown between the two groups with regard to anterior–posterior flexion of the trunk (mm) major in BG with respect to CG (27.85 ± 32.60 mm for BG vs. 20.02 ± 10.94 mm for CG, *p* = 0.001); pelvic inclination (mm; 0.84 ± 5.58 mm for BG vs. −1.17 ± 3.11 for CG, *p* = 0.018) decreased in BG with respect to CG; and twist of half-pelvis (degree) decreased in BG with respect to CG (−0.04 ± 2.99° for BG vs. 1.12 ± 1.75° for CG, *p* = 0.05); normalized lumbosacral inversion point (mm) decreased in BG with respect to CG (−0.96 ± 0.04 mm for BG vs. 0.99 ± 0.02 for CG, *p* = 0.005); surface rotation (amplitude; degree) major in BG with respect to CG (13.24 ± 8.19° for BG vs. 11.79 ± 3.92° for CG, *p* = 0.048); and lateral deviation (mm) major in BG with respect to CG (8.31 ± 6.71 mm for BG vs. 5.60 ± 3.38 mm for CG, *p* = 0.050; Table 2). The minus sign indicates a rotation to the left side. Compared with the values for the stabilometry test with EO and EC, a statistically significant difference was observed, respectively, for ellipse length (mm; 2364.39 ± 287.84 mm for BG vs. 1505.01 ± 330.29 mm for CG, *p* = 0.036) and ellipse area (mm^2^; 2789.40 ± 693.05 mm^2^ for BG vs. 2134.33 ± 281.89 mm^2^ for CG, *p* = 0.042) with EO and in ellipse area (mm^2^) with EC (2948.07 ± 1856.94 mm^2^ for BG vs. 2028.67 ± 538.19 mm^2^ for CG, *p* = 0.048; Tables 3 and 4) increased in BG with respect to CG for both conditions (EO and EC).

**Table 2.** Spine Rasterstereography: Trunk Values (Means and Standard Deviations)

Spine rasterstereography: Trunk values	Healthy group, mean ± SD	Breast cancer group, mean ± SD	*p* < 0.05
Anterior–posterior flexion (mm)	20.02 ± 10.94	27.85 ± 32.60	0.001
Lateral flexion (mm)	0.60 ± 12.67	−3.40 ± 24.12	0.378
Pelvic inclination (mm)	−1.17 ± 3.11	0.84 ± 5.58	0.018
Twist of half-pelvis (degree)	1.12 ± 1.75	−0.04 ± 2.99	0.005
Pelvis rotation (degree)	0.22 ± 3.51	1.38 ± 4.84	0.703
Apex of dorsal kyphosis (mm)	−0.36 ± 0.04	−0.33 ± 0.67	0.117
Thoracolumbar inversion point (mm)	−0.62 ± 0.04	−0.62 ± 0.05	0.564
Apex of lordosis (mm)	−0.79 ± 0.03	−0.78 ± 0.05	0.071
Lumbosacral inversion point (mm), normalized	0.99 ± 0.02	−0.96 ± 0.04	0.005
Cervical arrow (Stagnara; mm)	52.98 ± 13.62	54.56 ± 23.36	0.113
Lumbar arrow (Stagnara; mm)	35.92 ± 12.04	42.33 ± 9.71	0.250
Kyphotic angle (max; degree)	52.53 ± 8.25	54.69 ± 9.79	0.274
Lordotic angle (max; degree)	48.10 ± 7.54	51.45 ± 11.47	0.396
Surface rotation (+max; degree)	5.10 ± 3.98	5.21 ± 5.36	0.871
Surface rotation (−max; degree)	−6.68 ± 2.48	−8.02 ± 5.64	0.099
Surface rotation (amplitude; degree)	11.79 ± 3.92	13.24 ± 8.19	0.048
Lateral deviation (mm)	5.60 ± 3.38	8.31 ± 6.71	0.050
Surface rotation D4 (degree)	3.12 ± 2.28	4.42 ± 3.48	0.511
Surface rotation D4 (mass; degree)	−3.70 ± 5.28	−5.16 ± 9.61	0.195
Surface rotation D4 (amplitude; degree)	5.26 ± 3.30	9.64 ± 6.35	0.448

Variables were tested for normality using Shapiro–Wilk test; all the outcome measures were not normally distributed and so Mann–Whitney *U*-test was used to detect difference between groups.

**Table 3.** Stabilometry Parameters (Means and Standard Deviations), Open Eyes

Parameters (EO)	Group	Mean ± SD	*p* < 0.05
Cop minimum swings (mm)	BG	0.15 ± 0.16	0.377
	CG	0.10 ± 0.08	
Cop maximum swings (mm)	BG	17.42 ± 6.31	0.404
	CG	17.10 ± 9.77	
Transversal axis (mm)	BG	17.67 ± 8.34	0.073
	CG	14.84 ± 5.53	
Longitudinal axis (mm)	BG	29.64 ± 9.57	0.748
	CG	26.05 ± 10.13	
Ellipse length (mm)	BG	2364.39 ± 287.84	0.036
	CG	1505.01 ± 330.29	
Ellipse area (mm^2^)	BG	2789.40 ± 693.05	0.042
	CG	2134.33 ± 281.89	

Variables were tested for normality using Shapiro–Wilk test; all the outcome measures were not normally distributed and so Mann–Whitney *U*-test was used to detect difference between groups.

BG, breast cancer group; CG, control group of 30 healthy volunteer women.

**Table 4.** Stabilometry Parameters (Means and Standard Deviations), Closed Eyes

Parameters (EC)	Group	Mean	*p* < 0.05
Cop minimum swings	BG	0.18 ± 0.42	0.116
	CG	0.08 ± 0.06	
Cop maximum swings (mm)	BG	20.92 ± 9.57	0.673
	CG	17.64 ± 8.84	
Transversal axis (mm)	BG	21.35 ± 13.29	0.670
	CG	19.40 ± 14.29	
Longitudinal axis (mm)	BG	33.98 ± 13.77	0.322
	CG	27.27 ± 12.72	
Ellipse length (mm)	BG	1636.97 ± 532.62	0.615
	CG	1631.28 ± 348.82	
Ellipse area (mm^2^)	BG	2948.07 ± 1856.94	0.048
	CG	2028.67 ± 538.19	

Variables were tested for normality using Shapiro–Wilk test; all the outcome measures were not normally distributed and so Mann–Whitney *U*-test was used to detect difference between groups.

## Discussion

Our results demonstrate that postural alterations can be identified even after total mastectomy with external breast prostheses or tissue expanders. BG patients showed a greater limitation in sagittal spine alignment for anterior–posterior flexion of the trunk and lumbosacral inversion point more than a major pelvic inclination and twist of half-pelvis in favor of CG and an increase in surface rotation and lateral deviation. Also, in breast cancer patients, the postural control is carried out with greater energy expenditure considering the increasing of length and area with respect to the ellipse for the stabilometry evaluation.

Another study found that the amount of change in spinal alignment in postoperative breast cancer patients was significantly smaller with immediate breast reconstruction, compared with patients who received only unilateral mastectomy without reconstruction; thus, immediate breast reconstruction positively affects spinal alignment, leading to better posture and physical function.^31^ Our data are consistent with the literature and show that postural changes are evident even after surgical reconstruction during the first 12 months in chronic phase in breast cancer survivors: moreover, the postural alterations are not only on the sagittal plane of the column but also on the coronal plane and frontal one: it could be confirmed with the hypothesis that this three-dimensional alteration also with pelvic angle modification in the BG is linked to a greater variability compared to the postural balance for the stabilometric test with respect to CG. It would be possible to deduce that breast cancer survivors engage specific postural adaptations after surgery that suggest to be not functional for the postural control.

Breast reconstruction after mastectomy has an impact on proper body posture^32^ but postural imbalance in breast cancer survivors may result a complex interaction between biomechanical alterations of posture and also, other factors as peripheral neuropathy for chemotherapy.^33^

We hypothesize that an increase in trunk flexion in the BG, both with an alteration in pelvic inclination and twist of half-pelvis (Table 2), justifies the plus energy expenditure by BG for good postural control with respect to CG.^34^ In fact, for the stabilometry test, the BG results indicate greater ellipse length and ellipse area in the EO condition and higher ellipse area in the EC condition with respect to CG (Tables 3 and 4). Conversely, our results did not show a difference in the rotation of the trunk or lateral deviation with respect to the operated side in BG: some patients tend to have a closed posture toward the operated side due to shyness,^35^ pain, or tissue retraction with an increase in flexion of the trunk. We surmise that the alterations in other posture parameters, such as lumbosacral inversion point, lateral deviation, and surface rotation, during the *spine rasterstereography* test represent compensation of the body due to the appearance of spine sagittal curves that each patient achieves for herself as an ergonomically optimal body position.^36^

It is important to emphasize that none of these patients had yet undertaken a postural or upper limb rehabilitation program at the beginning of our study. In the literature,^37^ researchers often describe the efficacy of early rehabilitation after surgery for breast cancer and preoperative fitness,^38,39^ but in medical practice, this approach still happens infrequently. In fact, our patients were sent to the physiatric consultation for rehabilitation by their oncologist or their surgeon, after an average of 4.65 ± 3.30 months since surgery often the delay is also due to the patient's inability to face the chemotherapy and/or radiotherapy path together with the rehabilitation.

Our results should encourage physicians to consider early and personalized rehabilitative programs, and in particular, with attention to postural disorders and postural imbalance.^13^

Above all, the programs should not only focus on the recovery of upper limb function but also include exercises for realignment of the trunk and exercises for improving postural control.

According to Barbosa Jde et al.,^40^ the pelvis and trunk of women who have undergone quadrantectomy show better alignment compared with women who have been subjected to mastectomy but with little difference (90° vs. 91.3°) in the short period after surgery. Also, the women who underwent surgery on the side breast had shoulder elevation and ipsilateral inclination of the trunk, but follow-up of this group after completion of treatment is needed to determine the long-term postural changes.^40^ Other groups, such as Saggini et al.,^41^ reported a modification in posture and a significant increase in sway area after body mass alterations following mammoplasty, noting that posture and stabilometry data returned to equilibrium after 1 year. Their data suggest that posture control relies, at least in part, on feed-forward and feedback strategies.^41,42^ After treatment of breast cancer, 82.3% of women demonstrate faulty body posture without a significant relationship between the quality of body posture and oncological treatment,^43,44^ but other groups, based on different data regarding radiotherapy, have reported more shoulder dysfunction in patients who undergo radiation therapy.^45^

### Weaknesses and strengths

Our research expands on and enriches studies on posture and balance in women after breast surgery. There remain little data in the literature and virtually nothing on the use of specific tests, such as stabilometry and spine rasterstereography (with good objectivity). Above all, our results can be useful in developing more targeted rehabilitation programs that consider the recovery of not only the upper limb but also postural habit after breast cancer surgery. A limitation of the study is the lack of a postural assessment before surgery: for this reason it was considered a comparison group of healthy women. Our patients had never performed rehabilitative treatment and these data could be useful to investigate any changes that will be induced by the treatment itself at follow-up.

## Conclusion

Breast cancer survivors after prostheses or tissue expanders for mastectomy showed an adaptation of body posture and postural control and they engaged in specific postural compensation. A misalignment of the spine is present both on the sagittal plane, both on the coronal and frontal plane increased in BG with regard to anterior–posterior flexion of the trunk, surface rotation, and lateral deviation. Moreover, it is associated with a greater energy expenditure for the postural balance control increased in BG with respect to CG with a major ellipse area in EO and EC conditions and major ellipse length in EC condition.

## Abbreviations Used

BG: breast cancer group
BMI: body mass index
CG: control group of 30 healthy volunteer women
CMS: Constant–Murley score
CoP: center of pressure
DASH: Disability of the Arm, Shoulder, and Hand
DL: left lumbar dimple
DR: right lumbar dimple
EC: closed eyes
EO: open eyes
ITL: thoracolumbar
LLA: lumbar lordotic angle
MRC: Medical Research Council Manual Muscle Testing
SD: standard deviation
VAS: visual analog scale
VP: Vertebra prominens
